# Public opinion communication mechanism of public health emergencies in Weibo: take the COVID-19 epidemic as an example

**DOI:** 10.3389/fpubh.2023.1276083

**Published:** 2023-11-09

**Authors:** Siguo Ren, Chao Gong, Chen Zhang, Chen Li

**Affiliations:** ^1^School of Journalism and Communication, Wuhan University, Wuhan, China; ^2^Research Center for Japanese Studies, Tsinghua University, Beijing, China; ^3^College of Liberal Arts, Journalism and Communication, Ocean University of China, Qingdao, China; ^4^School of Management, Shanghai University of Engineering Sciences, Shanghai, China

**Keywords:** COVID-19, public health emergencies, Weibo public opinion, mechanism of transmission, spreading mechanism of public opinion

## Abstract

As a major public health emergency, the COVID-19 epidemic not only has a real risk of infection, but also easily generates public opinion risks. Under the condition of social communication of microblog, how to effectively identify public opinion and the harm of public health emergencies, avoid the overlay of real risk of epidemic and network public opinion risk, and prevent and resolve major public opinion risks is an important public opinion research topic in the new era. Taking the most influential outbreak of COVID-19 pneumonia in China in 2020 as an example, this paper discusses the effect of sudden major public health cases on Chinese microblogs and the spreading mechanism of public opinion. This paper mainly explores the communication motivation of public opinion from the four communication elements of the microblog public opinion center, public opinion object, public opinion carrier and public opinion ontology. And combined with the life cycle theory, this study analyzes the interaction between the communication elements of public opinion in different stages. In the fluctuation period of public opinion, the amount of public opinion information decreases relatively, and the frequent occurrence of secondary public opinion in the outbreak period promotes the continuation of public opinion. Compared with the diversified demands of public materials and medical assistance during the pandemic, public opinion in the fluctuation period reacts on the epidemic situation, which to some extent alleviates the tension of the epidemic situation. Based on this, this study puts forward the guidance strategy of public opinion of public health emergencies.

## 1. Introduction

The Corona Virus Disease 2019 (COVID-19) is a serious public health emergency. In addition to the characteristics of common public health emergencies, such as publicity, suddenness, complexity and continuity, it also has serious harm and wide impact. It not only seriously endangers the life and health of the public and the safety of property but also has a lot of negative impact on the normal production and life of the society. In the prevention and control of the epidemic, we have found that public health emergencies not only pose a threat to people’s physical environment but also lead to changes in people’s psychological environment, inducing public panic, and are likely to trigger derivative events and generate public opinion risks (1). China is undergoing a period of economic and social change, with variously intertwined interests and complicated society. In the prevention and control of the epidemic, in addition to taking effective measures to protect the life and property of the public, we should pay close attention to the social psychology and the public attitude and opinions, respond to and channel public opinion in a timely manner, and strive to prevent public opinion risks in an all-round way.

The research on public health emergencies in foreign countries began in the 1990s, slightly earlier than in China. Precious research in China often analyzed the impact of media reports on public health emergencies and proposed governance suggestions and strategies such as public opinion monitoring, public opinion warning, and public opinion guidance. At the same time, using microblog and public health as the subject words, there were 628 relevant studies in Chinese, including public health emergencies (294), COVID-19 (111), online public opinion (96), public health events (76), social media (59), COVID-19 epidemic (51), emergencies (44), health communication (39), online rumors (37), emotional analysis (34), public emergencies (32), mainstream media (26), new media (25), epidemic prevention and control (25), government microblog (24), influencing factors (24), governance research (24), opinion leaders (23), new government media (19), information dissemination (19), agenda setting (19), public opinion guidance (19), influencing factors research (18), Sina Weibo (16), People’s Daily (15), public health (15), communication effect (14), online public opinion (14), content analysis (13), epidemic information (13), microblog public opinion (13), communication mechanism (12), novel coronavirus pneumonia (12), social network analysis (12), information release (12), response strategies (11), public health crisis (11), Weibo text (11), and evolution of public opinion (10).

The current research mainly focuses on public health emergencies, the COVID-19, online public opinion, and social media. To sum up, many studies on COVID-19’s public opinion mainly focus on clustering analysis of public opinion articles and exploring the dissemination content of public opinion. The spread of public opinion in COVID-19 is more complex, and there are relatively few studies on the reasons for the spread of public opinion in COVID-19. The interaction between the components of public opinion spread also needs further exploration. Therefore, this study takes the outbreak of novel coronavirus pneumonia, which has the strongest influence in China in 2020, as an example to discuss the effect of public health emergencies on China’s microblog and the mechanism of public opinion diffusion. This paper mainly explores the communication motivation of public opinion from the four communication elements of COVID-19 microblog public opinion center, public opinion object, public opinion carrier and public opinion noumenon. And combined with life cycle theory, this study analyzes the interaction between elements of public opinion dissemination at different stages.

## 2. The transmission drivers of microwave public opinion on COVID-19

### 2.1. The transmission elements of the microblog epidemic of COVID-19

Although the formation, transmission and diffusion of Weibo public opinion are not simple linear information transmission models, they are very similar in the combination of transmission elements. In view of the composition conditions of Weibo public opinion, scholars generally hold three elements, four elements or five basic elements currently (2). The three elements of public opinion communication are generally composed of public opinion subject, public opinion object and public opinion carrier. The four elements theory adds the public opinion ontology and the five elements theory adds the public opinion environment. In this study, public opinion subjects and public opinion objects with the greatest influence in the communication of public opinion on Weibo of the novel coronavirus outbreak are selected for research. For example, in the early days of the novel coronavirus, there was a widespread online rumor that Shuanghuanglian could effectively inhibit the growth of the virus.

#### 2.1.1. Diversified Weibo users have become the main body of public opinion

The first is the public. The public is the main source of public opinion, and the characteristics of netizens in the public opinion of the COVID-19 microblog are more complex than those in the public opinion of any previous emergencies. Some scholars divide the public in public opinion into core stakeholders and non-core stakeholders, and the degree of stakeholder’s relevance is related to the participation of stakeholders in public opinion. Core stakeholders are often the source of information in public opinion, but the number of these people is not enough to form a large public opinion (3–5). In contrast, in the public opinion about COVID-19, there are no such onlookers with no interest, all the people are stakeholders. The core stakeholders are those whose lives, health and safety are directly damaged or seriously threatened and their relatives and friends. In the early stages of the COVID-19 pandemic, core stakeholders were the source of most information about the pandemic. In the subsequent diffusion of public opinion, the information disclosed by core stakeholders on Weibo was more likely to attract attention because they were in the center of the epidemic storm (6–9). The threat to their lives and health prompted them to make comments, ask others for help or give warnings.

The second is the government. In the public opinion on COVID-19, the government is the main provider of epidemy-related information, and the government’s policies and actions are directly related to the treatment and response of the COVID-19 outbreak, thus affecting the popularity of public opinion. When policies or actions of government meet the needs and demands of the public, the public opinion will gradually fade.

Finally, the media. The media has been a constant presence in Weibo public opinion on COVID-19, but its fragmented information is not enough to meet people’s needs for COVID-19 information. Market-based media paid attention to and reported news and information about the novel coronavirus before the epidemic was fully paid attention to and official media were on alert. Then there are the opinion leaders. When more and more online public opinions were aggregated on the microblog platform, the concept of microblog opinion leaders came into being. Buying Shuanghuanglian has been going on for a long time on Weibo. People who do not know the truth still believe that Shuanghuanglian can curb the novel coronavirus.

#### 2.1.2. Public health emergencies become public opinion objects

A public opinion object is an event or topic that causes public opinion. The event itself has the attribute that gives rise to the generation of public opinion. Kuang Wenbo put forward the storm formula of network public opinion communication, holding that the importance, fuzziness, sensitivity and accessibility of communication objects in network public opinion are positively correlated with the duration, heat and influence of public opinion storm (10–15). In the dissemination of public opinion, the popularity of smart phone terminals and the application of social media software have enhanced the accessibility of information. The COVID-19 epidemic endangers everyone’s life and health and has a direct impact on the public. Work stoppage and school suspension caused by COVID-19 also affect the livelihoods of countless people, as well as social and economic operations. The importance and sensitivity of such a major public health emergency is self-evident. In the early stage of the epidemic, the transmission channels, prevention and suppression methods, the development of the epidemic and other information were not clear enough, and it also took time for professionals to investigate the novel coronavirus, which led to the panic of the public in the face of the vague information and promoted the continuous growth of public opinion information (16–20).

### 2.2. The main causes of the spread of public opinion on Weibo of COVID-19

#### 2.2.1. Living needs

Human needs are the fundamental driving force of social development. Maslow put forward that survival needs are the basis of all other needs through his research. Only after meeting the basic needs such as survival and life, will people further pursue higher needs. In this sudden public health emergency, the COVID-19 pandemic is affecting people’s needs for survival. To prevent and control the spread of the novel coronavirus, the government first closed all channels of departure from Wuhan, and then other provinces and cities successively implemented quarantine and lockdown policies, closing roads and isolating people in communities and villages. After the lockdown policies, supplies were in short and prices skyrocketed in some parts. Especially in Wuhan, local people were caught off guard by the sudden lockdown, and they went to supermarkets to buy goods to ensure their survival and living needs under the quarantine (21). Survival and living materials were in short supply for a time. Based on the need for survival, the panicked people expressed their voices on Weibo, hoping that the government could guarantee the material needs under the isolation and obtain material assistance from all walks of life. When this demand is not met, it will become a continuous driving force, which drives people confined to a small area to express their demands through the Internet. In addition to material needs, there is also a need for medical treatment. At the peak of the epidemic, the increase of COVID-19 cases once exceeded the capacity of Wuhan hospitals. Hospital beds and other medical resources were in short, and it was difficult to find a single sickbed. The need for survival and medical supplies is a driving force for public opinion (22–24).

#### 2.2.2. Security requirements

As the fundamental driving force for social development, need has another characteristic, that is, the characteristic of never being satisfied. When old needs are met, new needs are created, or in other words, potential needs are stimulated. When mentioned “On Weibo, I post/forward information on COVID-19 to alert others to the risks of the epidemic.” 70% of the people said “very agree” or “somewhat agree.” People are willing to give warnings to remind others to take precautions against the perceived health risks. In the case of the COVID-19 epidemic, if the danger of survival does not exist, people begin to yearn for a stable and safe living environment and enter the pursuit of a higher level of safety needs (25–30). The new confirmed cases and deaths in the notification have stirred public sentiment. Making people feel that their lives and health are in immediate danger has stoked a sense of panic and raised concerns about health risks. To satisfy needs, many people still choose to buy Shuanghuanglian. It fully shows that the people’s sense of security is at stake.

#### 2.2.3. Expression of empathy

Public opinion is not all from the interests of demand and lack of motivation, but also from the need to love and belong to the formation of power. Maslow believed that survival and safety needs were caused by people’s deficit in adapting to the environment, and they were a kind of deficient motivation. Whether these needs could be satisfied depended on the external environment to a certain extent, and the need for love and belonging was beyond the actual needs of individuals. Thus, it was a transcendent motivation. Thousands of people prayed for Wuhan, which has been hit hard by the epidemic. Donations of materials and special industries still in operation are contributing to the fight against the epidemic. This need for love and care has made netizens follow the progress of the epidemic and prompted netizens to express their support for the epidemic prevention and fight.

### 2.3. The object causes of the spread of Weibo public opinions on COVID-19

Public opinion and the events that lead to it often show a resonance of the same frequency. The progress of the novel coronavirus epidemic has also affected the evolution of public opinion. The reason why the COVID-19 outbreak has been prolonged and high fever is closely related to the nature of the epidemic itself.

#### 2.3.1. The COVID-19 epidemic itself is of great harm

The COVID-19 outbreak is arguably the most influential public health emergency since the beginning of the 21st century. The strong infectivity of the novel coronavirus threatens everyone’s life, health and safety, and the lockdown policy under the epidemic is also affecting people’s normal life. Daily reports of new cases and deaths are rattling nerves. In addition, the epidemic has had an impact on people’s normal life. People must be isolated at home, and work and life are directly affected. Many businesses have been hit by lockdown and quarantine policies, and numerous small businesses and economies have gone out of business during the pandemic.

#### 2.3.2. The COVID-19 epidemic is of interest to the people of the whole country

The higher the degree of public interest in an event or topic is, the higher the probability that people will devote their energy and time to participate in the event. However, the attention and content of public opinions about the novel coronavirus outbreak on Weibo are unprecedented, because the novel coronavirus outbreak is an event that concerns the interests of people all over the country, and its interests even involve the international community (31). People are concerned about how many people have been infected, how many have been cured, how many have died, whether there have been any cases in their area, when the outbreak will be brought under control and when the quarantine will be lifted. All kinds of information related to the public have prompted people to ask questions and learn about the COVID-19 outbreak.

## 3. Mechanism of action among factors of public opinion communication in Weibo on COVID-19

### 3.1. The evolution process of Weibo public opinion on COVID-19

#### 3.1.1. Latency of public opinion: slow growth of information

**Figure 1:**
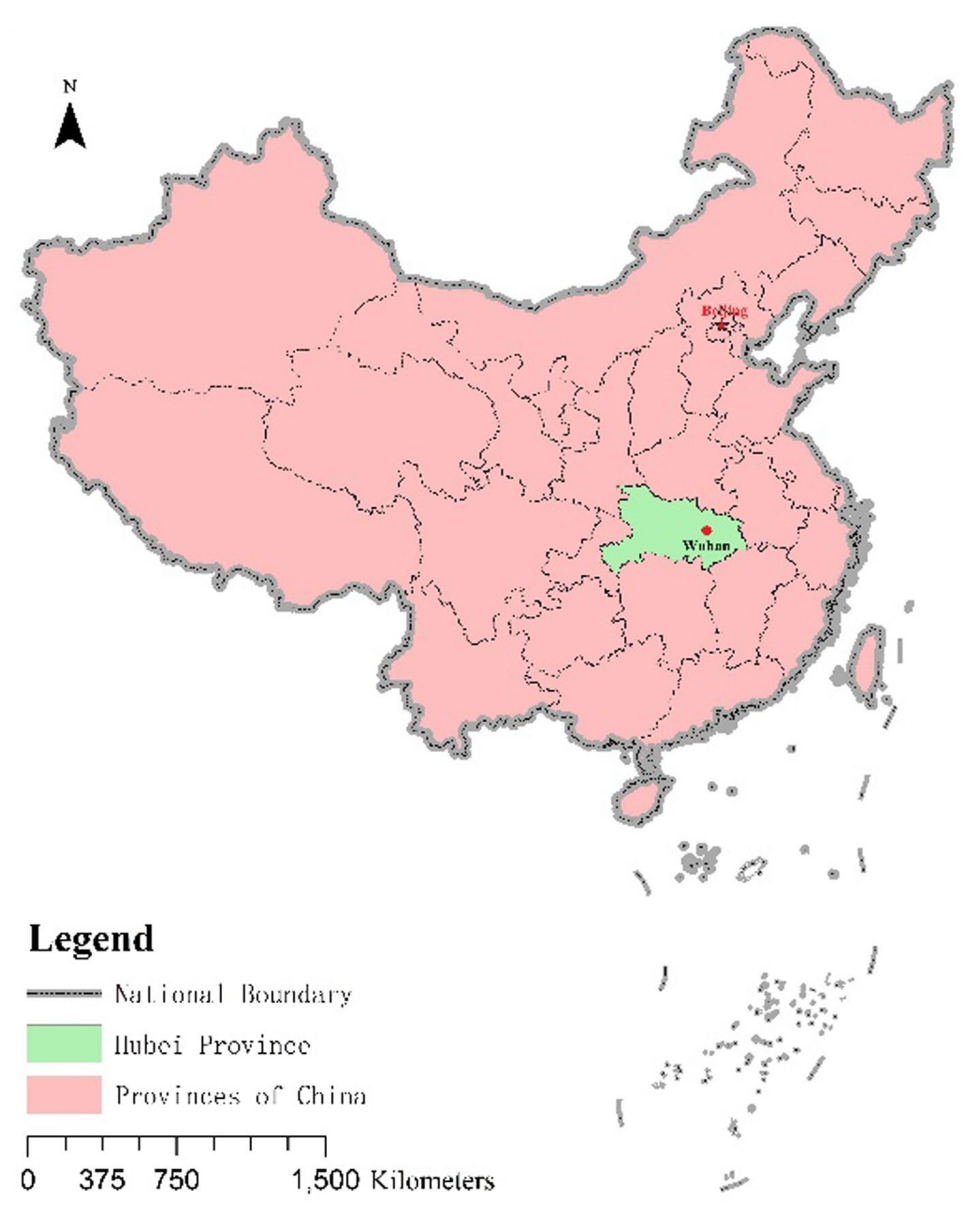
The location of Wuhan in China.

The incubation period (2019.12.30–2020.1.19) is the stage in which the novel coronavirus has emerged, the scale of public opinion has not been formed yet, and a small number of people have been informed and disseminated of relevant information. The outbreak of the novel coronavirus pneumonia happened earlier than public opinion. The pneumonia cases appeared in early December, but did not attract attention, and the epidemic was still in the latent stage. It was not until the number of cases gradually increased and Wuhan began to pay attention to them that local people began to panic and public opinion began to sprout (Figure 1). However, since no obvious human-to-human transmission was found in Wuhan pneumonia and the Wuhan pneumonia of unknown cause could not be confirmed as SARS, the public thought that the situation was not serious, and the public opinion did not heat up for the time being. According to the monitoring information on the website, the information about Wuhan unknown pneumonia has not increased significantly, but new information has been generated and spread. But as the number of cases and deaths rose, the public became alarmed. Netizens began to question and inquire through Weibo posts.

#### 3.1.2. Outbreak period: information outbreak growth

The outbreak period (2020.1.20–2020.1.28) is the period when the amount of public opinion information shows an explosive growth and the attention of public opinion continues to rise. At this time, the emergency has been known by the public and caused extensive discussion. On January 20, Zhong Nanshan delivered the message of human-to-human transmission of the novel coronavirus through a press conference, which confirmed the seriousness of the novel coronavirus. Since then, the state media has been focusing on the issue from the top down, with continuous screen-swiping coverage. Since then, 31 regions have launched major public health emergency response. The consequences of the spread of the virus caused by the movement of people in the early stage of the epidemic have begun to emerge. The emergence of new cases in various regions has made people feel the threat of the epidemic, and people’s travel, work and life under the epidemic have also been affected. First, at 10 am on January 23, the Wuhan pneumonia virus prevention and Control headquarters, which was caused by the novel coronavirus in Wuhan, notified the closure of all roads and the suspension of traffic operations in the city, causing panic among the people in Wuhan. At the same time, the spread of the epidemic began to appear, and other provinces also began to implement epidemic prevention and control. On the same day, Hangzhou, Guangdong and Hunan provinces started the first response of a serious public health emergency, while Shaanxi, Heilongjiang, Inner Mongolia, Urumqi and Gansu provinces also confirmed their first cases, pushing public opinion to its first peak. On January 24, the number of diagnosed cases in the province exceeded 1,000, followed by the first response of major public health emergencies in various regions. The impact of the epidemic is gradually spreading to people in all provinces (32–35). As of January 28, the cumulative number of confirmed cases of the novel coronavirus pneumonia in China exceeded 5,000. Both the number of infected people and the speed of transmission of the virus exceeded that of SARS, and the public opinion reached the highest peak.

#### 3.1.3. Fluctuation period: peak evolution

Entering the fluctuation period of public opinion (2020.1.29–2020.3.11), the attention of public opinion passed the peak and once showed negative growth. However, the influence of public opinion was still huge in terms of the number of public opinions, and then there were several stages of growth. At this stage, the popularity of public opinion continues. However, with the progress of public opinion events and related topics derived, the growth rate of public opinion information keeps changing, and the evolution of public opinion presents a phenomenon of multi-peak fluctuation. It takes time to study COVID-19, the object of public opinion generated by the COVID-19 public opinion event and other emergencies. Medical experts need time to study the symptoms, treatment and vaccine research of COVID-19, and the storm of public opinion will not stop while the results of the research are not available and new COVID-19 cases are still emerging everywhere.

#### 3.1.4. Fading period: information is continuously reduced

**Figure 2:**
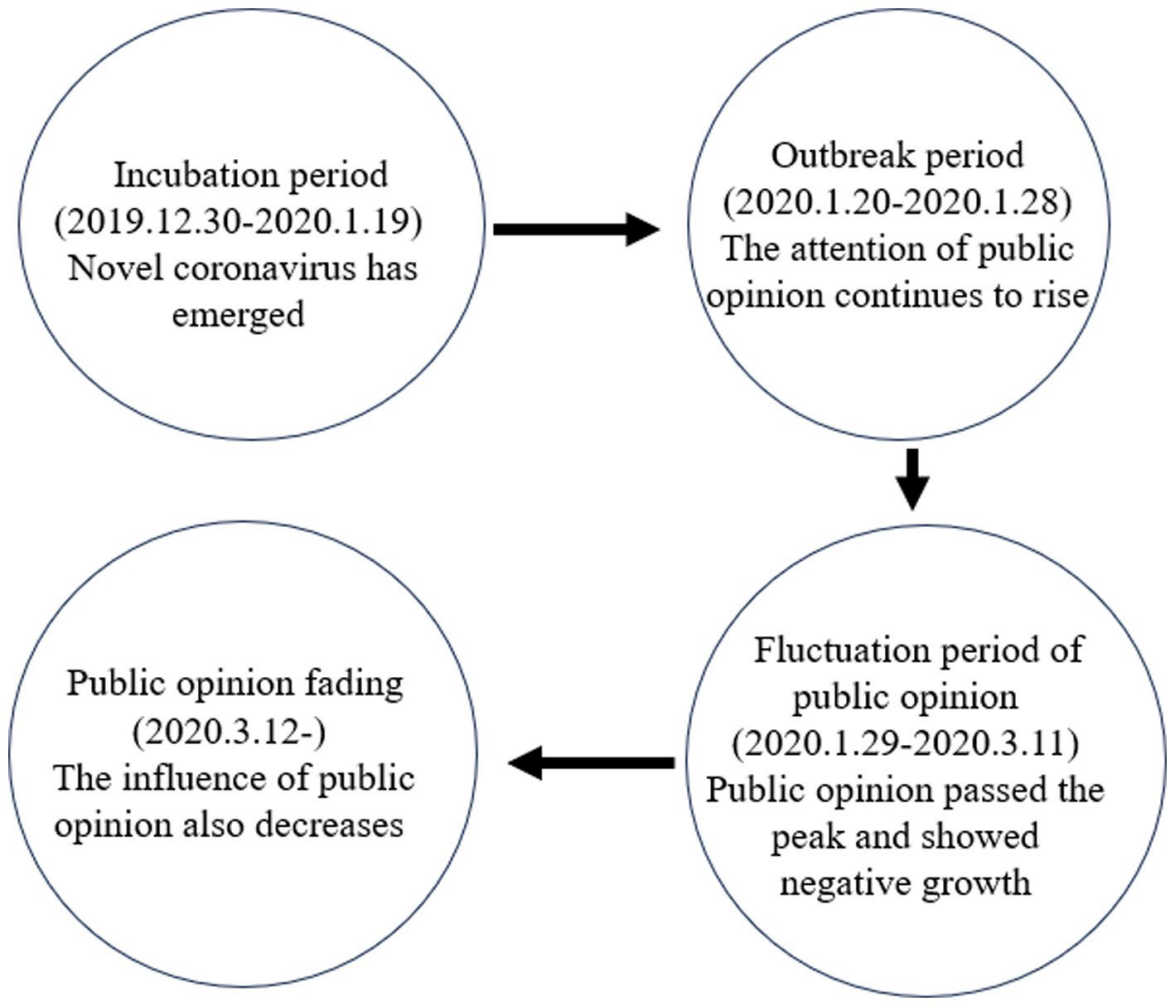
The evolution process of Weibo public opinion on COVID-19.

The period of public opinion fading (2020.3.12) refers to the period when the attention of public opinion continues to decline, the amount of public opinion information gradually decreases, and the influence of public opinion also decreases (Figure 2). At this stage, the object of public opinion was gradually brought under control, and the generated public opinion and secondary public opinion also came to an end. On February 18 and February 19, the number of newly cured and discharged cases in the whole province and Wuhan reached the number of newly cured and discharged cases in all parts of the country, respectively. The Committee of the Joint Prevention and Control Organization of the General Office of the State Council issued Several Opinions on Scientific, Reasonable and Accurate Measures for Regional Prevention and Management of COVID-19, which also began to stipulate the resumption of work and production. Since February 21, cities have lowered the level of major public health emergencies and gradually lifted travel restrictions. The effects of combat and prevention are already there. By March 11, the rise of the epidemic had been halted, with new cases down to single digits and stable for a week. Regional governments are also updating disease risk levels and phasing out the first level of response to a serious public health emergency.

### 3.2. The host-object interaction of public opinion latency

#### 3.2.1. Social weakening of risk affects subjects’ risk perception

The risk perception of the public directly affects the direction of the benign or malignant development of the existing public opinion. The social amplification theory of risk holds that after the occurrence of a risk event, the risk will be amplified or weakened through media processing and social communication, and the attitude and decision made by the public under the perception of risk amplification or weakening often have a new impact on the risk event in turn. During the incubation period of Weibo public opinion on the COVID-19 epidemic, the perceived risk of most people is smaller than the actual technical risk. At the end of December 2019, news of the outbreak of unknown pneumonia in Wuhan became a hot search on Weibo, and the media also circulated relevant reports from Wuhan. At this time, many people also heard the news from social media and interpersonal communication, but the public did not get the correct information (36–40). Due to technical and administrative concerns in the early stages of the outbreak, Wuhan did not communicate the seriousness of the outbreak to the outside world. Wuhan City announced that there was no apparent human-to-human transmission of Wuhan pneumonia. On January 1, 2020, the Weibo Safe Hubei also issued an announcement, saying that eight people had been found spreading untrue information about Wuhan pneumonia, which made the public not aware of the risk of the novel coronavirus outbreak in a timely way.

#### 3.2.2. Risk perception affects the behavioral decisions of public opinion subjects

The spread of disease is influenced by the willingness of the population to adopt preventive public health behaviors, which is significantly related to its risk perception. The free movement of people under the inaccurate perception of risk has, on the contrary, facilitated the carrying and spreading of the virus, further expanding the actual technical risks of the COVID-19 outbreak. At the level of information space, the inconsistencies between Wuhan officials and the public in conveying information, as well as the media’s inability to speak words, led to the decline of the public’s vigilance. As can be seen from the amount of information monitored by the Weiwei event, the COVID-19 outbreak in Wuhan did not attract the attention of the public before January 19, and only a small number of people participated in the release and dissemination of information about the COVID-19 outbreak in Wuhan. Most of the crowd ignored the news amid the rumor-mongering and refutation. On the physical space level, people flow freely without any defense, and people’s perceived risks often have consequences on actual risks through behavioral decisions. The outbreak originally appeared in the South China seafood market in Wuhan. But since January 19, a series of COVID-19 cases have been reported in Shenzhen, Guangdong, Beijing and other large cities with high mobility, followed by other provinces and cities such as Chengdu, Sichuan and Tianjin. Early personnel without control and protection of personnel flow has brought bad results. The lack of attention and vigilance caused by too little risk perception further expanded the scope of the epidemic and increased the risk of the epidemic (41–44). It was not until January 12 that Zhong Nanshan confirmed the existence of human-to-human transmission of the novel coronavirus, which aroused the attention of the whole country from top to bottom and started the prevention and fight against the epidemic from a top-level strategy.

### 3.3. Main-ontology interaction during public opinion outbreak

#### 3.3.1. Media reports meet the information needs of the public

After the rise of social media, agenda-setting theory was once considered as a scene that had lost its function. In fact, it displayed new content under the new communication pattern. At first, the traditional agenda-setting theory proposed by McCombs believed that mass media determined the topics of communication. Later, the traditional agenda-setting theory evolved into the attribute agenda-setting theory, that is, the media not only had great power in the setting of the topics, but also could influence the audience to think about the topics from what angle. Under the impact of new communication technology and communication pattern, the media’s ability to set the topic is indeed weakened. However, the professionalism and authority of media in the interpretation of issues still exist, which determines that media can set the interpretation angle of issues through news interpretation. The angle of media coverage under the COVID-19 epidemic largely leads the direction of public opinion. The People’s Daily and other mainstream media’s main agenda is to report on the topic of helping Wuhan to fight the epidemic. When the government was fully aware of the seriousness of the novel coronavirus outbreak and public opinion was still boiling, the media began to attach importance to the issue from top to bottom and occupied every major channel of news circulation through comprehensive and multi-angle screen-based reports (45, 46). In terms of popular blog posts on Weibo platforms, those published by official media such as People’s Daily, CCTV.com and Beijing News are the most popular and influential.

#### 3.3.2. The government’s epidemic response measures affect the special epidemic plan

In the face of the epidemic, the public always pays close attention to the government’s response measures to the epidemic. The government, as a policy maker and social manager, not only plays a leading role in the response to public health emergencies, but also plays an important role in the public opinion of public health emergencies. It occupies the third place among the retweets of popular microblogs, with an average retweet of about 30,000 times. The government’s response to the epidemic is an important node affecting the evolution of public opinion and can affect the turn of public opinion. During the outbreak of public opinion, there are two important measures that affect the evolution of public opinion. According to the public opinion chart, on January 20, the public opinion information of the novel coronavirus epidemic on Weibo changed from a gentle development to a rapid growth, with a sudden increase in the trend of public opinion.

## 4. Strategies and suggestions for public opinion guidance of public health emergencies in Weibo

### 4.1. Restructuring the governance order of Weibo space

Firstly, have a rational understanding of the public space on Weibo. The dissemination of information in Weibo space is dominated by an “absolute minority” of key nodes, with a large proportion of entertainment industry celebrities in the key nodes (47). There is a huge difference between Weibo space and Habermas’ public space. In the process of spreading public emergencies on Weibo, apart from the core nodes, there has not been an authoritative information release point, nor has there been a true “opinion leader.” The grassroots consciousness of political participation has risen, but its influence is not enough. Therefore, the public space on Weibo still needs to be guided in a standardized and orderly manner. Secondly, it is recommended to have pre-installed self-media monitoring mechanisms such as Weibo. Establish a joint and several liability systems for platforms, users, and other parties, especially for sudden public events. The monitoring mechanism should be moved forward to ensure that the operation, information release, topic setting, and discussion of Weibo space platforms operate in a standardized manner within the legal framework. Thirdly, pay attention to the diversified governance and cultivation of “key minority” in Weibo space. Strengthen the public communication character of the three subgroups of “communication amplifier,” “information provider,” and “communication bridge,” and encourage “key minority” to participate in the governance of Weibo space after sudden public events. The government should guide mainstream media to speak widely, especially local media Weibo and stakeholder media Weibo in the location of the incident, to form a public opinion leading force.

### 4.2. Balancing the timeliness of information sources and media authority

The sources of information include not only frontline journalists, but also the public as individuals. The Internet era has activated individuals’ ability to manipulate social communication resources, forgotten information needs and preferences, and various micro resources (idle time, knowledge, and experience, etc.). The result of this activation is the reconstruction of the media ecosystem, greatly improving individual discourse expression and content production capabilities. There are generally two ways for individuals to participate in public discourse expression (48–58). One is to be more proactive in expressing individual opinions, which are mostly scattered. Another approach is to integrate with news producers and become one of the main producers of news, breaking the boundaries of news production in the era of individual dissemination. In the process of epidemic reporting, the role of a journalist is no longer just an observer. After the closure of Wuhan, frontline reporters had dual identities as Wuhan residents and journalists, presenting a dual perspective of participants and observers in the report. Due to professional news training, importance and timeliness are prioritized in selecting topics and perspectives. The original news consumers have become news producers who provide first-hand information and self-expression, and the information they provide often has an advantage in timeliness. Professional journalists have a more sensitive sense of news and standardized news operations. Cultivating the news literacy of the public and combining the professionalism of journalists with the timeliness of user information sources may produce more high-quality and popular news works.

### 4.3. Using rumor refutation platforms to effectively block rumors

The current emergency reporting procedures basically cover the information and topics to be informed by the public in case of sudden public safety incidents. However, as the cost of spreading rumors decreases, rumors during the epidemic are not conducive to the implementation of prevention and control work, nor is it conducive to the emotional mobilization and public opinion direction of the public in fighting against the epidemic. The establishment and timely updates of rumor refutation platforms provide ideas for blocking rumors in the era of social media. In the early stages of the epidemic, people were isolated at home and spent more time obtaining epidemic related information from social media. Due to the explosive growth of information, it is difficult to distinguish authenticity, and many rumors have spread on social media, leading to an overload of public information and an increase in anxiety. Inviting experts to refute rumors by the media and labeling rumors on social media platforms have played a positive role in curbing rumors and quelling anxiety. The fact verification technology of the media and the authoritative judgment of medical experts contribute to the construction of a refutation platform. The media industry already requires verifying news facts, verifying news sources, and evaluating news credibility. This coincides with the refutation mechanism. The spread of rumors can cause public panic and anxiety, breeding social instability factors. In the process of curbing rumors, on the one hand, the media should ensure the standardization of the reporting and verification process and ensure the accuracy of news reporting facts and refutation facts. On the other hand, relying on the news fact verification mechanism of the media and the professional judgment of medical experts, the investigation results and key points can be published based on evidence to refute rumors. On social media platforms, blocking is better than dredging, and the verification process and reasons should be publicly disclosed to society.

### 4.4. Establish a unified management system for public opinion supervision

Public opinion supervision is a very important form of social supervision. In this incident, public opinion supervision played a good role in promoting the open and transparent operation of public power departments. But at the same time, it should also be noted that there may be deviations in public opinion supervision, and there may even be problems of using the name of supervision to show ill intentions. In major public health emergencies, the public is more likely to be disturbed by irrational emotions, so it is necessary to provide appropriate guidance for public opinion supervision. Establish a unified management system from mainstream media to various types of self-media, and promptly handle the real problems exposed by impartial media, and report the handling situation on various media platforms in a timely manner. For negative media under the guise of false supervision, regulations should be implemented.

### 4.5. Weibo users should abide by the law and do not believe or spread rumors

Weibo users shall not infringe upon the legitimate rights and interests of state organs, communities or organizations when exercising their rights and interests of independent speech according to law. It is forbidden to publish and discuss political and religious issues, form an information chain that spreads rumors, deceive people to spread rumors, make subjective assumptions, and casually repost or comment on Weibo that may cause controversy. You are also not allowed to publish hearsay or unconfirmed information on your micro blog, vent your personal feelings, and denigrate or insult others in other ways.

## 5. Conclusion

After entering the 21st century, the occurrence of sudden public health accidents is more and more frequent. Regarding the prevention of public health emergencies, as a major issue in China, public opinions about public health emergencies in China also frequently appear on Chinese microblog websites. Public opinion about a public health emergency lasts as the event lasts. This paper also makes an in-depth analysis of the causes of public opinion communication from the four major factors of public opinion communication subject, public opinion communication object, public opinion communication carrier and public opinion communication ontology, and gives countermeasures for public opinion guidance in sudden public health accidents. The investigation of Weibo public opinion on the COVID-19 epidemic in China can enhance the risk communication of public opinion on sudden public health accidents, and thus better mitigate the risk of the epidemic.

Weibo is a very popular social media platform that provides a place for people to share, discover and discuss Chinese content. Here are some possible mechanisms for calibrating content on Weibo: (1) Review mechanism: Weibo can set up a review mechanism to review all published content. Reviewers can check whether the content conforms to the platform regulations and whether there are violations, such as publishing false information and malicious attacks on others. If the content is deemed problematic, the reviewers can decide whether to allow the content to be published. (2) Reporting mechanism: Users can report the content posted by other users, indicating that the content has problems or violations. The platform will review the reports and if the content is identified as problematic, it will deal with it, such as deleting or banning the publication. (3) Community norms: Weibo can develop community norms that clearly specify the rules that users should abide by on the platform. Community norms can include prohibiting the Posting of illegal, obscene, violent, false and other content, or they can stipulate that users should respect others and not engage in malicious attacks. Users need to follow these guidelines when Posting content. (4) User feedback: Weibo can set up a user feedback mechanism to allow users to evaluate the content posted by other users. These reviews can reflect the content’s quality, usefulness, or compliance with platform regulations. The platform can process the content accordingly based on user feedback. (5) Technical means: Weibo can use technical means to calibrate the content. For example, the use of natural language processing technology for text sentiment analysis, language style analysis, etc., to determine whether the content conforms to the platform regulations. Images can also be reviewed using image recognition technology to determine whether they contain illegal content. These mechanisms can be used individually or in combination to achieve calibration of content on the microblogging platform. However, it is important to note that these mechanisms need to be supported and cooperated by users in order to be truly effective.

In recent years, the communication pattern has been changing constantly, and the public opinion formed by short videos has become more and more prominent. This paper has made a certain analysis of short videos from the perspective of content drive, but there is still room for further exploration. With the update of communication technology and the reform of communication pattern, the public opinion of public health emergencies still needs further research in the new communication pattern.

## Data availability statement

The original contributions presented in the study are included in the article/supplementary material, further inquiries can be directed to the corresponding authors.

## Author contributions

SR: Writing – original draft. CG: Writing – original draft. CZ: Writing – review & editing. CL: Writing – review & editing.
